# The peroxisomal importomer can accommodate an intrinsically disordered protein of 1247 residues

**DOI:** 10.1007/s00418-026-02483-9

**Published:** 2026-05-12

**Authors:** Marc P. Pedersen, Arjen M. Krikken, Rinse de Boer, Ida J. van der Klei

**Affiliations:** Molecular Cell Biology, Groningen Biomolecular Sciences and Biotechnology Institute, University of Groningen, Groningen, the Netherlands

**Keywords:** Yeast, Peroxisome, Protein translocation, Nsp1

## Abstract

Peroxisomal matrix proteins are nuclear encoded and synthesized in the cytosol. It is well established that folded, cofactor-containing, and even oligomeric matrix proteins can pass the peroxisomal membrane. Here, we studied whether relatively long unfolded proteins can be imported into peroxisomes using the yeast *Hansenula polymorpha* as a model organism. First, we designed fusion proteins containing parts of the intrinsically disordered N-terminal region of 630 residues of *Saccharomyces cerevisiae* nucleoporin 1 (Nsp1). These included fusion proteins containing the first N-terminal 301 or 601 residues or two times the 601 residues region of Nsp1. Green fluorescent protein was added at the N-terminus, and a peroxisomal targeting signal 1 at the C-terminus. Fluorescence microscopy revealed that all three fusion proteins colocalized with a peroxisomal marker protein, indicating that large unfolded protein domains can be imported into peroxisomes. To obtain a fully unfolded protein, we replaced the folded GFP tag by a double human influenza hemagglutinin tag, which unlike GFP is unable to fold. An in vivo protease protection assay showed that a portion of the produced proteins localized to peroxisomes, which was confirmed by quantitative immuno-electron microscopy analysis. On the basis of our observations, we conclude that a fully unfolded protein of over 1200 residues (almost 500 nm in length) can pass the peroxisomal membrane.

**Supplementary Information:**

The online version contains supplementary material available at 10.1007/s00418-026-02483-9.

## Introduction

Sorting of peroxisomal matrix proteins involves a peroxisomal targeting signal (PTS). The most common PTS is the dodecamer sequence PTS1 located at the extreme C-terminus of peroxisomal matrix proteins (Gould et al. 1989; Osumi et al. 1991; Brocard and Hartig 2006). The PTS1 is recognized by a soluble receptor protein, Pex5. Pex5 also can bind a PTS3, which is formed by scattered residues in the cargo polypeptide and is only formed upon folding of the cargo (Kempinski et al. 2020). *Saccharomyces cerevisiae* contains a second PTS1 receptor, Pex9. Pex9 is a condition-specific PTS1 receptor and recognizes only a subset of PTS1 proteins (Effelsberg et al. 2016; Yifrach et al. 2016). Relatively few proteins have a PTS2, which is present close to the N-terminus and recognized by the receptor protein Pex7.

The first step of PTS1 import is recognition of the newly synthesized PTS1 protein by the soluble Pex5 receptor protein in the cytosol (Gould et al. 1989; Osumi et al. 1991). Next, the receptor–cargo complex docks to the peroxisomal membrane. Recent studies indicated that subsequently the Pex5–cargo complex fully traverses the peroxisomal membrane through a transient pore, which is formed by Pex13 (Skowyra et al. 2022; Gao et al. 2022; Gaussmann et al. 2024). Interestingly, the pore-forming structure of Pex13 resembles the natively unstructured mesh domains that occur in the nuclear pore-forming complex (NPC) (Skowyra et al. 2022; Gao et al. 2022; Ravindran et al. 2023). The PTS1 cargo subsequently dissociates in the peroxisomal matrix. Pex5 export to the cytosol occurs through a small rigid channel consisting of the peroxins Pex2, Pex10, and Pex12, which forces Pex5 to unfold and release the cargo (Feng et al. 2022a, b; Pedrosa et al. 2023). The AAA-ATPases Pex1 and Pex6 are required for adenosine triphosphate (ATP)-dependent export of Pex5 across the peroxisomal membrane to the cytosol.

Most proteins are transported across a membrane in an unfolded state (e.g., the translocase of the outer membrane (TOM) and translocase of the inner membrane (TIM) complexes in mitochondria and the Sec translocon of the endoplasmic reticulum (ER)) (Gemmer and Förster 2020; Pei and Dalbey 2022; Busch et al. 2023). However, some protein transport machineries can accommodate folded proteins. These include protein import through the NPC and the twin‐arginine protein transport (TAT) mechanism of chloroplasts and bacteria (Patel et al. 2014; Frain et al. 2019; Paci et al. 2021).

Several studies have shown that the peroxisomal importomer also can accommodate folded or even cofactor-containing or oligomeric proteins (Baker et al. 2015; Kalel and Erdmann 2018; Bürgi et al. 2023). Moreover, Pex5-dependent PTS3 import relies on folding of the cargo protein prior to protein translocation (Kempinski et al. 2020). Studies in the yeast *Hansenula polymorpha* have shown that alcohol oxidase (Aox) is only imported into peroxisomes upon folding and binding of its cofactor flavin adenine dinucleotide (FAD). Interestingly, Aox remains targeted to the peroxisomes even upon removal of the PTS1, as long as its cofactor, FAD, is properly bound (Gunkel et al. 2004; Ozimek et al. 2006). After import in the peroxisomal matrix, the FAD-containing Aox monomers dissociate from the Pex5 receptor and oligomerize into enzymatically active octamers. Also, import of *H. polymorpha* peroxisomal catalase (Cta) is blocked when it contains a point mutation that specifically affects heme binding and tetramerization. This mutant Cta protein mislocalizes to the cytosol, despite the fact that it contains a PTS1. This result indicates that proper folding, cofactor binding, and tetramerization of Cta is crucial to translocate the protein across the peroxisomal membrane (Williams et al. 2012). These data support the view that protein folding and cofactor binding is important for the peroxisomal matrix protein import machinery, similar to the TAT translocon (Frain et al. 2019; Rudowitz and Erdmann 2023). Interestingly, it has previously been demonstrated that, in mammalian cells, the peroxisomal matrix import machinery has a preference for Pex5 bound to monomeric cargo rather than oligomeric. This is the case for acyl-coenzyme A (CoA) oxidase 1 and urate oxidase (Freitas et al. 2015).

Nonbiological components have also been successfully targeted to the peroxisomal matrix. Gold particles with diameter in the range of 4–9 nm coated with PTS1 peptides were found to localize inside peroxisomes (Walton et al. 1995). Also, multiconjugated Traptavidin-PTS1 particles coupled to mCherry (619 kDa, 12.5 nm diameter) were successfully imported into peroxisomes (Yang et al. 2018). Such nonbiological components obviously do not require folding. Thus, the peroxisomal importomer is remarkably flexible and allows for the import of various artificial components and particles.

Although we now know that folded proteins can cross the peroxisomal membrane, we do not know whether peroxisomal proteins must fold to pass the transient import pore.

**Fig. 1:**
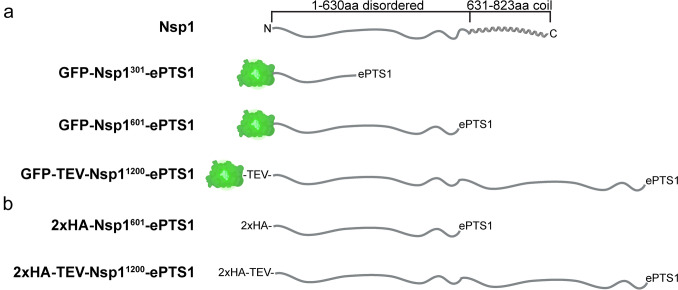
Schematic representation of all Nsp1 domain-containing fusion proteins used in this study. **a** Schematic representation of the GFP constructs fused to intrinsically disordered regions of *S. cerevisiae* Nsp1 at the N-terminus. Full-length Nsp1 consists of a 630-amino-acid intrinsically disordered region and a coiled-coil region from residue 631 to 823. Nsp1^301^ consists of the first 301 amino acids, Nsp1^601^ is the unfolded Nsp1 region, and Nsp1^1200^ consists of two times the 601-amino-acid, intrinsically disordered region of Nsp1. The Nsp1^1200^ construct has a Tobacco Etch Virus (TEV) nuclear-inclusion-a (NIa) endopeptidase cleavage site between GFP and Nsp1. Green protein cartoons indicate GFP. **b** Overview of the human influenza hemagglutinin (HA)-tagged constructs. A 2xHA tag was fused at the N-terminal 601 or 1200 residues of Nsp1. The Nsp1^1200^-containing fusion protein has a TEV NIa cleavage site inserted between the 2xHA tag and Nsp1^1200^ region. The C-terminus of all fusion proteins contained the relatively strong PTS1 (ePTS1) “ASLTDGVEKSKL” (de Vries 2008)

In this study, we investigated whether long unfolded proteins can be imported into the peroxisomal matrix using the yeast *H. polymorpha*. For this purpose, we constructed fusion proteins consisting of (parts of) the intrinsically disordered N-terminal region of *Saccharomyces cerevisiae* nucleoporin 1 (Nsp1) (Dekker et al. 2023) (Fig. 1). Localization experiments by fluorescence and immuno electron microscopy (iEM) together with an in vivo protease protection assay showed that all constructs, including a fully unfolded polypeptide of 1247 residues (calculated molecular weight (MW) of 153.5 kDa, estimated length almost 500 nm), were able to pass the peroxisomal membrane. These observations indicate that the peroxisomal importomer can accommodate very long unfolded proteins.

## Materials and methods

### Strains and growth conditions

All *H. polymorpha* strains used in this study are listed in online resource Table S1. Yeast cells were cultivated at 37 °C in mineral medium (van Dijken et al. 1976) supplemented with 0.5% glucose (Sigma Aldrich, #14431-43-7), 0.5% methanol (Sigma-Aldrich, #67-56-1) or a mixture of 0.3% methanol and 0.1% glycerol (Sigma-Aldrich, #G7893). Leucine (Sigma-Aldrich #L8912) was added to a final concentration of 60 μg/ml, when necessary.

For imaging experiments, cells were grown on glucose to an optical density (OD_660_) of 1.0. For induction of the TEV protease Nia, cells were grown to the exponential growth phase on glucose (OD_660_ 1.8–2.0) and diluted to an OD_660_ of 0.1 in mineral medium (0.3% methanol and 0.1% glycerol). The OD_660_ was measured using a spectrophotometer (StaRRcol, #SC-60-S).

Selection of positive transformants was performed on YPD plates (1% yeast extract (Gibco, #2441197), 1% peptone (Gibco, #2024469), and 1% dextrose (glucose)) containing 100 μg/ml zeocin (InvivoGen #ant-zn-05), 100 μg/ml nourseothricin (Werner Bioagents/Jena Bioscience, #AB-102XL), and/or 300 μg/ml hygromycin (InvivoGen #ant-hg-5). *Escherichia coli* DH5α (Hanahan 1983) was used for cloning purposes, and selection was performed using LB plates (1% Bacto tryptone (Gibco, #211705), 0.5% yeast extract (Gibco, #212750), and 0.5% NaCl (Sigma-Aldrich, #S9888)) containing 100 μg/ml ampicillin (Sigma-Aldrich, #A9518-5G).

### Plasmids and molecular techniques

Plasmids used are listed in online resource Table S2. Primers used are listed in online resource Table S3.

For this study, the unfolded protein region of *S. cerevisiae* nucleoporin (Nsp1, #YJL041W, UniProt #P14907) was chosen. Three variants of the *Sc*Nsp1 genomic sequence, encoding the unfolded protein region of Nsp1, were created: 903 bp (half-length Nsp1, 301 aa), 1803 bp (full-length Nsp1, 601 aa), and 3600 bp (two full-length Nsp1 in sequence fused together, 1200 aa).

Plasmid pHIPN18 GFP-Nsp1^301^-ePTS1 was constructed using plasmid pHIPN18 mGFP. A Nsp1^301^ fragment consisting of 903 bp was produced by polymerase chain reaction (PCR) amplification using primers Nsp1-F and Nsp1-R300. These primers contain an *Nde*I restriction site, ePTS1, stop codon, and a *Xba*I restriction site. The corresponding PCR product and pHIPN18 mGFP were digested with *Nde*I (Thermo Scientific, #FD0583) and *Xba*I (Thermo Scientific, #FD0684) and ligated together. Plasmid pHIPN18 GFP-Nsp1^601^-ePTS1 was constructed with a similar procedure but using the Nsp1-R600 reverse primer instead, producing a 1809-bp fragment.

The GFP-TEV-Nsp1^1200^-ePTS1 plasmid was constructed by PCR amplification of two fragments and the pHIPN18 mGFP plasmid as a backbone. Firstly, a fragment containing an *Nde*I restriction site, a nine-nucleotide linker region, a *Bam*HI (Thermo Scientific, #FD0054) restriction site, TEV protease cleavage sequence, the 1809 bp of NSP1^601^, and a *Sal*I restriction site (Thermo Scientific, #FD0644) was produced using primers BamHI-TEV-NSP1-F and TEV-NSP1-SalI-R. Secondly, a fragment containing an *Nde*I restriction site, a nine-nucleotide linker region, a *Sal*I restriction site, the 1809 bp of NSP1^601^, the 1ePTS1 sequence, a stop codon, and a *Xba*I restriction site (NSP1-600-12SKL) was constructed using primers Sali-Nsp1-F and Nsp1-R600. These two fragments were combined using the *Sal*I restriction site and then ligated into *Nde*I and *Xba*I restriction enzyme-digested pHIPN18 mGFP backbone.

The 2xHA-Nsp1^601^-ePTS1 plasmid was constructed by DNA synthesis (GenScript) of the cloning plasmid pUC57 2HA-NSP1^601^-AarI. This plasmid contains a *Hin*dIII restriction site, a start codon, two copies of the HA tag, the first 178 nucleotides of Nsp1 and the *Aar*I restriction site and was digested with *Hin*dIII (Thermo Scientific, #FD0504) and *Aar*I (Thermo Scientific, #ER1581 and New England Biolabs, #R0745S). This fragment was ligated into *Hin*III + *Aar*I-digested GFP-TEV-Nsp1^1200^-ePTS1 to produce the 2xHA-Nsp1^601^-ePTS1 plasmid.

To construct plasmid 2xHA-TEV-Nsp1^1200^-ePTS1, a 938-bp fragment was produced by PCR amplification with primers 345.FW.2xHA and 346.RV.2xHA, using plasmid GFP-TEV-Nsp1^1200^-ePTS1 as a template. The 938-bp fragment was isolated and digested with restriction enzymes *Not*I (Thermo Scientific, #FD0593) and *Bam*HI and ligated into the plasmid GFP-TEV-Nsp1^1200^-ePTS1 digested with the corresponding enzymes to produce the 2xHA-TEV-Nsp1^1200^-ePTS1 plasmid.

Genes were expressed under control of alcohol oxidase 1 (AOX1), alcohol dehydrogenase 1 (ADH1), or endogenous promoter. For expression under control of the AOX1 and ADH1 promoter, the full-length gene was cloned. All plasmids were linearized and integrated into the genome as described before (Faber et al. 1994; Saraya et al. 2012). DNA restriction enzymes were used as recommended by the suppliers (Thermo Scientific or New England Biolabs). PCR for cloning was carried out with Phusion High-Fidelity DNA Polymerase (Thermo Scientific, #F530S). Colony PCR was carried out using DreamTaq DNA Polymerase (Thermo Scientific, #EP0701). For all DNA ligation, the Rapid DNA Ligation kit was used (Thermo Scientific, #K1423). For DNA sequence analysis, the Clone Manager 5 program (Scientific and Educational Software, Durham, NC) was used. For fluorescence microscopy (FM) localization studies, Pex3-mKate2 was used as peroxisomal marker.

### Biochemical methods

For trichloroacetic acid (TCA, Sigma-Aldrich, #T6399) protein precipitation (McCammon et al. 1994) of 3 OD_660_ units yeast culture, 10 mM ZnCl_2_ (Sigma- Aldrich, #208086) was added to the 12.5% TCA solution and to the 80% acetone (Supelco, #90872) wash solution to inhibit the activity of the yeast strains expressing the TEV protease NIa (Dougherty et al. 1989; Joseph and Savithri 2000). Samples were prepared in Laemmli buffer (Bio-Rad, 4xLaemmli #1,610747). Sodium dodecyl sulfate-polyacrylamide gel electrophoresis (SDS-PAGE) was performed using 7.5% discontinuous gels as described previously (Baerends et al. 2000) or using 4–15% gradient gels (Bio-Rad, 4–15% Mini-PROTEAN® TGX™ Precast Protein Gels, #456108). Equal amounts of protein were loaded per lane.

Semi-dry transfer (Thermo Scientific, Pierce G2 Fast Blotter) of proteins from the SDS gel to a nitrocellulose membrane (Amersham Protran 0.2 NC, #10600001) was performed as described previously (Kyhse-Andersen 1984) with the 60 min standard transfer time program. Blots were decorated with mouse monoclonal antiserum against GFP (Santa Cruz Biotechnology, Inc. #sc-9996), mouse monoclonal antiserum against anti-HA tag (Roche, #11583816001), or rabbit polyclonal antiserum against elongation factor 1 alpha (EF1α), which served as a loading control (Kiel et al. 2007). Secondary goat anti-rabbit immunoglobulin G (IgG) (Thermo Scientific, #31460) or goat anti-mouse IgG antibodies (Thermo Scientific, #31430) conjugated to horseradish peroxidase were used for detection.

For detection, chemiluminescent solution (Amersham ECL Prime, #RPN2232) was applied to the membranes according to the manufacturer’s guidelines (Bio-Rad, Chemidoc Imaging System). Blots were imaged using the Bio-Rad ChemiDoc Imaging System, quantified using Bio-Rad ImageLab software (version 6.1), and edited using Adobe Illustrator (version 28.7.1). The density of each band measured was standardized by dividing the density of the corresponding loading control band.

### Fluorescence microscopy (FM)

A Zeiss Axioscope A1 fluorescence microscope with a 63× 1.40 NA objective (Carl Zeiss), CoolSNAP HQ2 digital camera (Photometrics CoolSNAP HQ2, Birmingham, UK), and MICRO-MANAGER software (version 1.4) was used for capturing images. The GFP signal was visualized with a 470/40 nm bandpass excitation filter, a 495 nm dichromatic mirror, and a 525/50 nm bandpass emission filter. The mKate2 fluorescence was visualized with a 587/25-nm bandpass excitation filter, a 605 nm dichromatic mirror, and a 647/70 nm bandpass emission filter. ImageJ (version 1.53u) and Adobe Illustrator (version 28.7.1) were used for image analysis and editing. All FM images within each figure were processed similar unless otherwise stated.

Airyscan images were captured with a confocal laser scanning microscope (Carl Zeiss, LSM800) equipped with a 32-channel gallium arsenide phosphide photomultiplier tube (GaAsP-PMT), Zen 2009 software (Carl Zeiss), and a 63× 1.40 NA objective (Carl Zeiss) for oil immersion. The GFP signal was visualized by excitation with a 488 nm laser, and mKate2 was visualized with a 561 nm laser. Cells were imaged at room temperature. Fiji (version 1.53t) and Adobe Illustrator (version 28.7.1) were used for image analysis and editing. All Airyscan images within each figure were processed similarly.

For Airyscan imaging, cells were washed with 1× phosphate-buffered saline (PBS) (10 mM Na_2_HPO_4_ (Millipore, # 567550), 0.137 M NaCl (Sigma-Aldrich, #S9888), 0.27 mM KCl (Sigma-Aldrich, #P9541), and 0.18 mM KH_2_PO_4_ (Sigma-Aldrich, # P5655), then fixed in 1% formaldehyde (FA) (Sigma-Aldrich, #F8775) solution for 15 min on ice and washed in 1 × PBS prior to imaging. For the 1% (FA) solution, we prepared 16% (w/v) FA at 65 °C on a heating block and dilute 16× in 1 × PBS to make a 1% (w/v) FA solution (62.5 μL/1 mL PBS) (Krikken et al. 2020). Cells were imaged at room temperature.

### Electron microscopy (EM)

Immuno-EM (iEM) was performed as described previously (Thomas et al. 2018). For iEM, polyclonal antiserum raised against alcohol oxidase (αAox) (van der Klei et al. 1988) and catalase (Cta) (Keizer et al. 1992; Waterham et al. 1993) were used as previously described (Salomons et al. 2000). Labeling of HA was performed using monoclonal antibodies (Sigma-Aldrich H9658; 1:100 dilution), followed by goat anti-mouse IgG antibodies conjugated to 6-nm gold particles (Aurion, the Netherlands). For 12 peroxisomes, the distance of each gold particle to the peroxisomal membrane was measured using the line selection tool in Fiji (version 1.53t).

## Results

### A GFP-tagged structurally unfolded protein of 601 resides localizes to peroxisomes

To test whether unfolded proteins can be imported into *H. polymorpha* peroxisomes, we used fragments of nucleoporin 1 (Nsp1) from *S. cerevisiae* (Fig. 1). Nsp1 is a component of the NPC and consists of a long unfolded N-terminus containing phenylalanine–glycine (FG) repeats (residues 1–630) followed by a coiled-coil domain (residues 631–823) at the extreme C-terminus (Fig. 1) (Peyro et al. 2021; Fragasso et al. 2022; Dekker et al. 2023). The FG repeats make the N-terminal domain of Nsp1 disordered (Denning et al. 2003; Dekker et al. 2023).

**Fig. 2:**
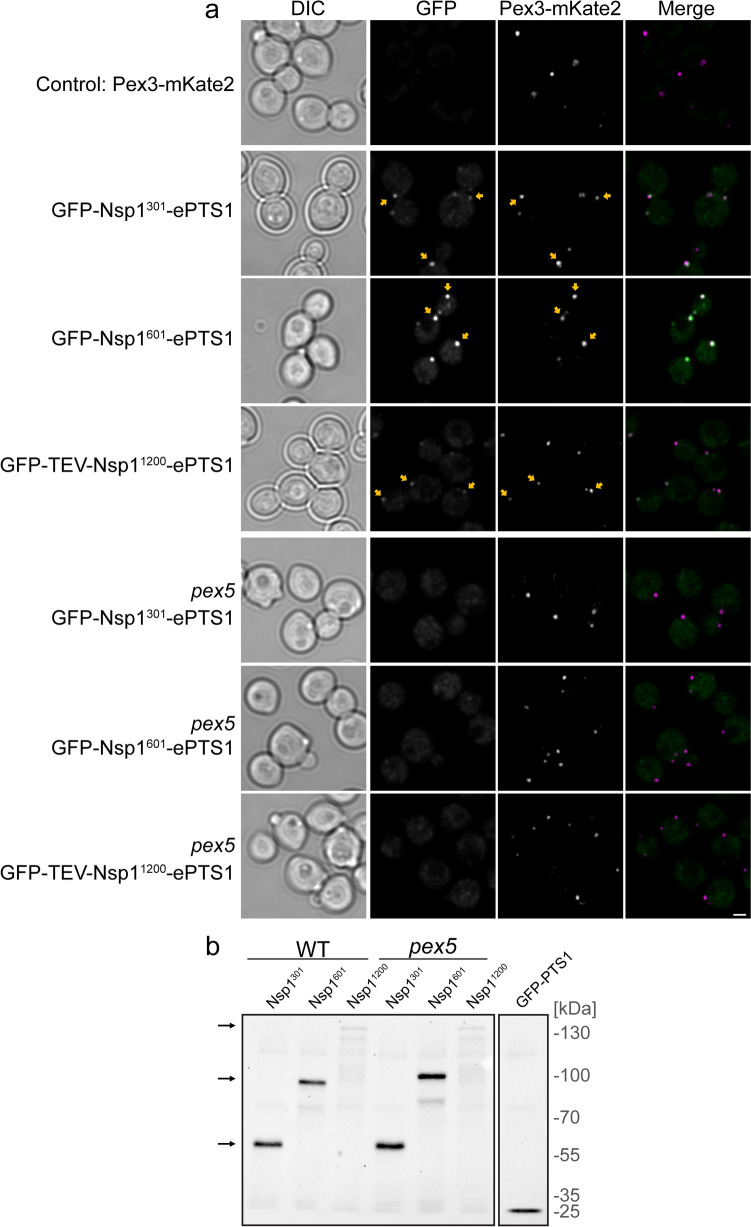
GFP-Nsp1 fusion proteins localize to peroxisomes. **a** Single-focal-plane confocal laser scanning microscopy (CLSM, Airyscan) images of glucose-grown *H. polymorpha* wild-type (WT) and *pex5* cells producing the indicated GFP fusion proteins. Pex3-mKate2 was introduced as peroxisomal marker. Control WT cells only produce Pex3-mKate2. Scale bars, 1 μm. *n* = 3 independent experiments. **b** Western blot analysis of whole-cell lysates using α-GFP antibodies. Black arrows indicate the position of the bands of GFP-Nsp1^301^-ePTS1 (calculated molecular weight [MW] 58.36 kDa), GFP-Nsp1^601^-ePTS1 (MW 90.24 kDa), and GFP-TEV-Nsp1^1200^-ePTS1 (MW 153.5 kDa). Equal amounts of protein were loaded per lane. A strain only producing GFP-PTS1 was used as control. [kDa] indicates the bands for the MW marker. FM images and blot shown represent three independent experiments (*n* = 3)

Three *H. polymorpha* strains were constructed, producing fusion proteins containing the first 301, 601, or two times the 601 N-terminal residue region of Nsp1 (Fig. 1a). GFP was fused to the N-terminus of these proteins to enable their localization by fluorescence microscopy (FM) (Fig. 1a). A relatively strong PTS1 (ASLTDGVEKSKL) was added at the extreme C-terminus of all three fusion proteins (ePTS1) (de Vries 2008; Brocard and Hartig 2006; Deloache et al. 2016). We designated these proteins as GFP-Nsp1^301^-ePTS1, GFP-Nsp1^601^-ePTS1, and GFP-TEV-Nsp1^1200^-ePTS1, respectively (Fig. 1a). Cells were cultivated on glucose and subjected to FM analysis (Fig. 2a). As shown in Fig. 2a, all three GFP fusion proteins showed a punctate pattern indicative of peroxisomal localization. The puncta represent peroxisomes as they colocalize with the peroxisomal membrane marker Pex3-mKate2. Invariably, also cytosolic GFP fluorescence was observed. The GFP puncta were not caused by bleed-through of the Pex3-mKate2 signal in the GFP channel (Fig. 2a).

GFP puncta were absent and only cytosolic fluorescence was observed when the fusion proteins were produced in cells of a *pex5* control strain, which lack the PTS1 receptor Pex5 (Fig. 2a).

Western blot analysis of whole-cell lysates using α-GFP antibodies revealed that all GFP fusion proteins were present at the expected molecular weight (MW) of the full-length fusion proteins (Fig. 2b). The band of the largest variant (GFP-TEV-Nsp1^1200^-ePTS1) was quite weak compared with the other two. In all cases, weaker bands of lower molecular weight were detected as well. Because the proteins are recognized by antibodies raised against GFP, these represent truncated variants of the fusion proteins, which lack an ePTS1 and therefore cannot be imported into peroxisomes.

### HA-tagged Nsp1 fusion proteins can also pass the peroxisomal membrane

**Fig. 3:**
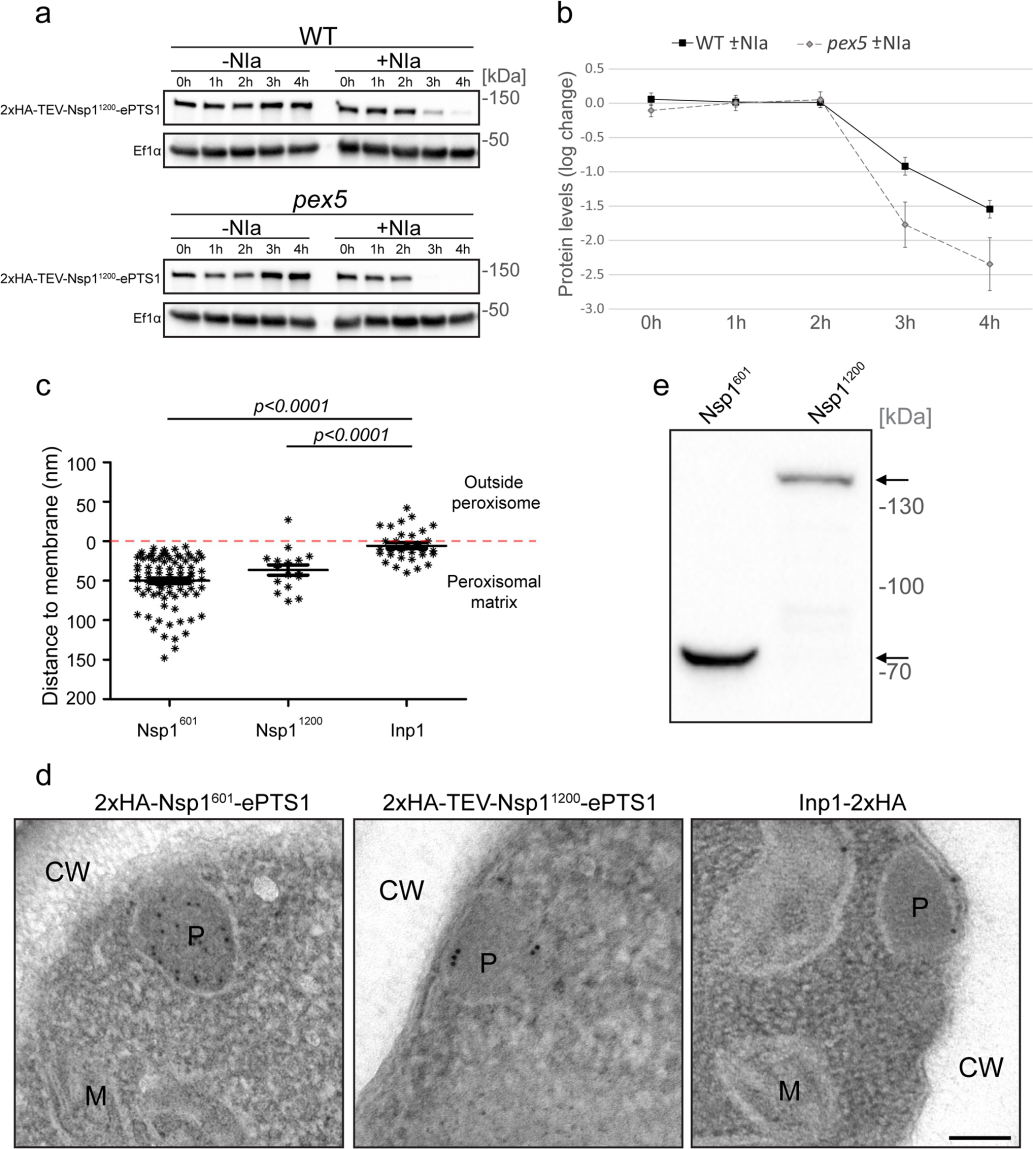
A fully unfolded protein is imported into peroxisomes. **a** Western blots of whole-cell lysates from WT and *pex5* strains without or with the 2xHA-TEV-Nsp1^1200^-ePTS1 fusion protein. The strains were precultivated in glucose medium (0 h) and shifted to glycerol/methanol medium. Samples were taken at the indicated time points. Equal amounts of protein were loaded per lane corresponding to 3 OD units of culture. The blots were decorated with αHA antibodies. αEf1α was used as loading control. Equal exposure times were used for the αHA and αEf1α blots, respectively. **b** Plots showing the log-transformed ratio of the level of the fusion protein in WT ± NIa or *pex5* ± NIa cells over time. **c** Quantitative measurements of distances between gold particles and the peroxisomal membrane in 12 peroxisomes. Two-tailed Student’s *t* test was performed. Error bar represents the standard error of the mean (SEM). **d** iEM localization in glucose-grown WT cells producing 2xHA-Nsp1^601^-ePTS1 (left panel), 2xHA-TEV- Nsp1^1200^-ePTS1 (middle panel), and P_*ADH1*_::Inp1-2xHA (right panel). CW; cell wall. P; peroxisome. M; mitochondria. Scale bar, 200 nm. **e** Western blots of lysates from cells used in **d** using αHA antibodies. Equal amounts of protein were loaded per lane. Black arrows indicate the band localization for the 2xHA-Nsp1^601^-ePTS1 (65.29 kDa) and 2xHA-TEV-Nsp1^1200^-ePTS1 (128.28 kDa) corresponding to their expected MW. kDa indicate the MW bands for the marker. A representative blot of three independent experiments is shown (*n* = 3)

The GFP fusion proteins still contain a tertiary domain, because GFP folds into a β-barrel structure (Yang et al. 1996; Ormö et al. 1996). To obtain fusion proteins that are completely unfolded, we exchanged the GFP tag with two times the human influenza HA tag (2xHA) (Fig. 1b). The HA tag is a small peptide consisting of nine amino acids, which do not fold into any tertiary structure (Green et al. 1982; Wood 2014). Since the 2xHA tagged protein cannot be detected by FM, we used an established in vivo protease protection assay (Faber et al. 2001) to monitor import. A fusion protein was constructed containing the 2xHA tag followed by a TEV NIa protease cleavage site (2xHA-TEV-Nsp1^1200^-ePTS1, Fig. 1b) and was produced under control of the constitutive P_*ADH1*_. The NIa protease was produced under the control of the methanol-inducible *AOX* promoter (P_*AOX*_). The NIa protease does not contain a PTS and hence remains in the cytosol of *H. polymorpha* cells (Faber et al. 2001). WT cells were precultivated on glucose medium (which represses the P_*AOX*_) and shifted to glycerol/methanol medium to induce NIa expression. Time-resolved western blot analysis confirmed that the 2xHA tagged protein was present in the glucose grown inoculum cells (Fig. 3a; *T* = 0* h*). After 3 h and 4 h induction of NIa synthesis, the band intensities decreased, indicating that a portion of the fusion protein was cleaved. However, a band for 2xHA-TEV-Nsp1^1200^-ePTS1 could still be detected at these late time points, suggesting that a portion of the fusion protein was protected against cleavage by the cytosolic NIa protease (Fig. 3a). Import of the fusion protein was confirmed by the observation that, in *pex5* control cells producing 2xHA-TEV-Nsp1^1200^-ePTS1, the fusion protein became below the limit of detection 4 h after induction of NIa protease synthesis. In WT and *pex5* control cells that do not produce the NIa protease, the levels of the full-length fusion proteins did not decrease as expected (Fig. 3a).

Quantification of the western blots confirmed that the level of the fusion protein was invariably higher in WT cells compared with the *pex5* control cells after induction of NIa expression. As shown in Fig. 3b, the log ratio of the levels of the fusion protein in WT ± Nia expression was higher compared with *pex5* ± Nia expression at all time points. These data indicate that, in WT cells, a portion of the 2xHA-TEV-Nsp1^1200^–ePTS1 fusion protein localizes to the peroxisomal matrix, where it is protected against cleavage by the NIa protease.

Further evidence for import of fully unfolded fusion proteins was obtained by iEM using antibodies against the HA tag (Fig. 3c, d) and cells of strains producing 2xHA-Nsp1^601^-ePTS1 or 2xHA-TEV-Nsp1^1200^-ePTS1. We previously successfully performed iEM studies in *H. polymorpha* using the same antibodies (Wu et al. 2020). Figure 3d shows that both fusion proteins are present in the peroxisomal matrix. The labeling was relatively weak in cells producing the largest construct (2xHA-TEV-Nsp1^1200^-ePTS1), in line with the outcome of the NIa protection assay, which revealed that relatively low amounts of this protein are produced and protected against NIa cleavage (Fig. 3a). Western blotting using antibodies against HA showed that both proteins were present at the expected MW (65.29 kDa and 128.28 kDa, respectively) (Fig. 3e).

As a control, we performed iEM analysis of a *H. polymorpha* strain producing Inp1-2xHA, a protein that associates to the outside of the peroxisomal membrane via interaction with the membrane protein Pex3 (Krikken et al. 2020). Quantitative analysis of the localization of the gold particles showed that the gold particles are present at both sides of the peroxisomal membrane (distance < 50 nm), when cells producing Inp1-2xHA were used. However, both Nsp1 fusion proteins show a different localization pattern, which corresponds to the presence of the proteins in the peroxisomal matrix (Fig. 3c).

**Fig. 4:**
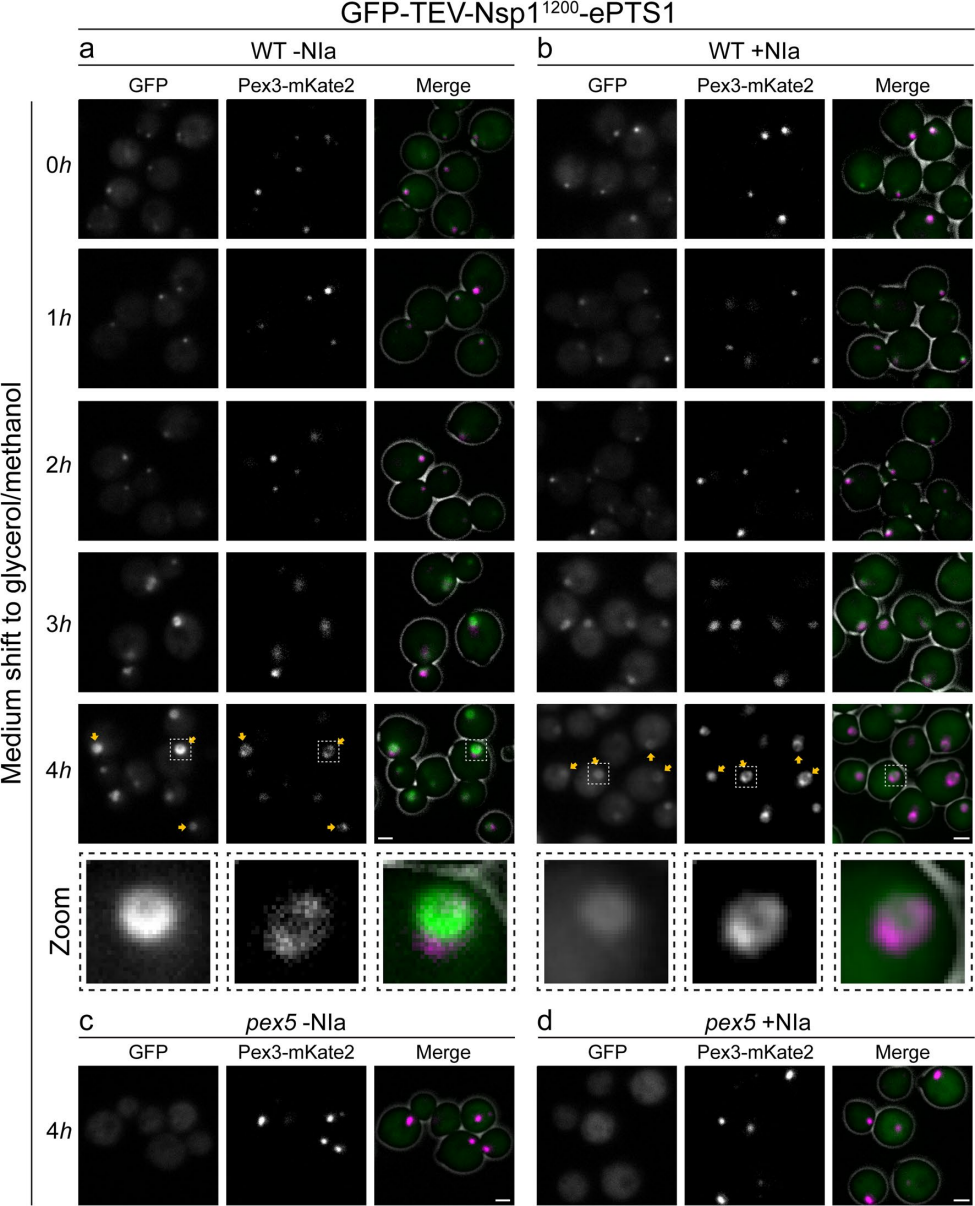
Cytosolic NIa does not affect peroxisomal matrix protein import. FM images of WT (**a**, **b**) and *pex5* cells (**c**, **d**) producing the GFP-TEV- Nsp1^1200^-ePTS1 fusion protein with (**b**, **d**) or without (**a**, **c**) Nia expression. The cells were precultured in glucose medium (0 h) and shifted to glycerol/methanol medium to induce NIa expression. Pex3-mKate2 was introduced as a peroxisomal marker. Yellow arrows indicate localization of the fusion proteins to the peroxisomes. In the lowest panel of **a** and **b**, higher-magnification images are shown of peroxisomes marked in the 4-h images. FM images are representative of three independent experiments (*n* = 3). Scale bar, 1 μm

As a control, to rule out that cytosolic NIa has an effect on peroxisomes/peroxisomal matrix import, we analyzed import of the largest fusion protein containing both GFP and a TEV site (GFP-TEV-Nsp1^1200^-ePTS1, Fig. 1a) by FM. Similar results were obtained for WT cells expressing NIa (Fig. 4b). Invariably, GFP fluorescence colocalized with the peroxisomal marker (Fig. 4a) at all time points before and after the shift to glycerol/methanol medium. Upon the shift to glycerol/methanol, the puncta increased in size owing to peroxisomal growth, as expected. These data indicate that the NIa protease has no impact on import of the fusion proteins. GFP fluorescence was not present in puncta in *pex5* control cells with or without NIa expression, as expected (Fig. 4c, d).

In summary, on the basis of the in vivo protease protection assay and quantitative iEM localization studies, we conclude that a portion of the HA-tagged Nsp1 fusion proteins is fully imported into the peroxisomal matrix and hence able to pass the peroxisomal membrane.

### Long unfolded PTS1 proteins do not obstruct the peroxisomal importomer

**Fig. 5:**
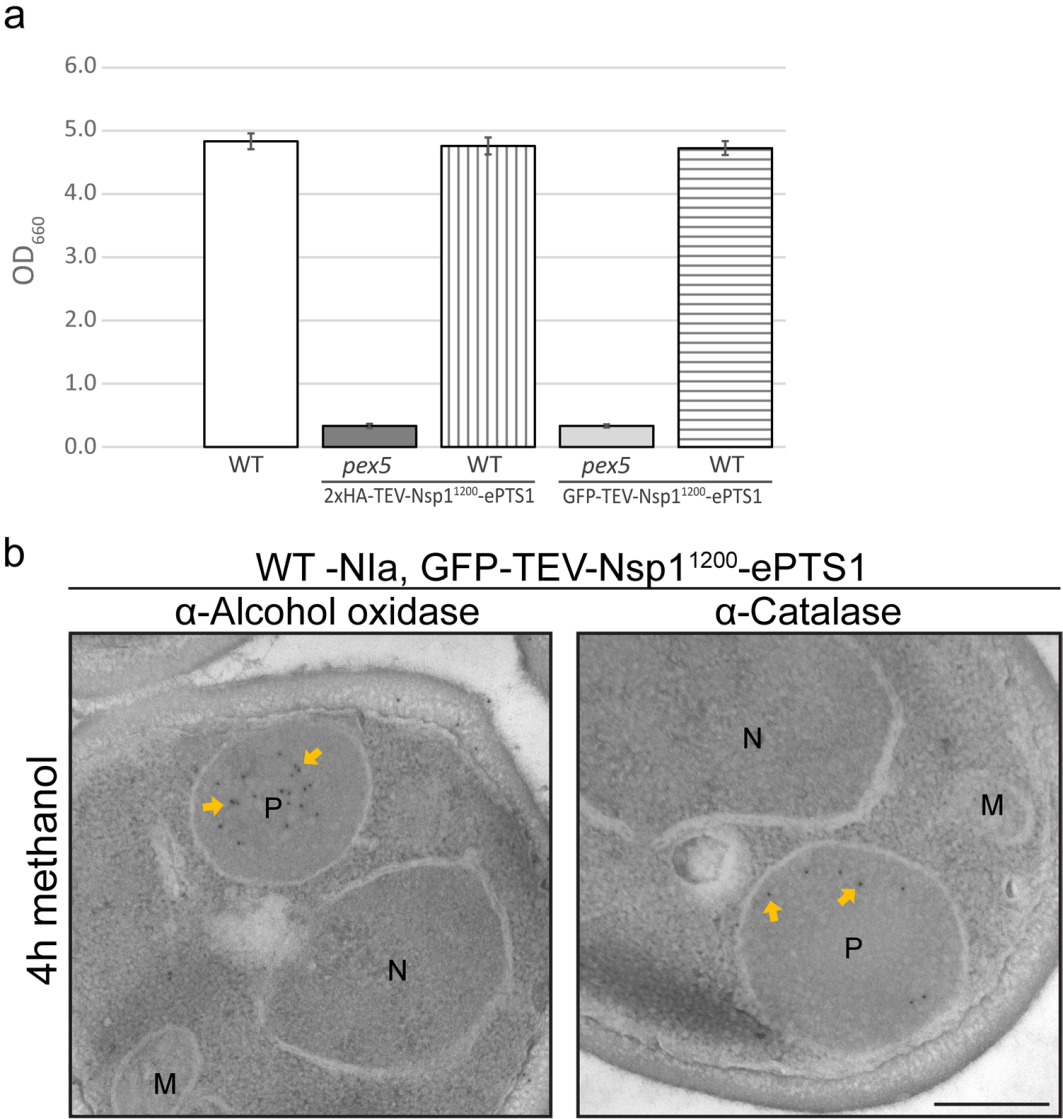
Import of a large, unfolded protein does not affect import of endogenous peroxisomal proteins. **a** WT control cells (white), WT expressing 2xHA-TEV-Nsp1^1200^-ePTS1 (vertical stripes) or GFP-TEV-Nsp1^1200^-ePTS1 (horizontal stripes), and *pex5* cells expressing 2xHA-TEV-Nsp1^1200^-ePTS1 (dark grey) or GFP-TEV-Nsp1^1200^-ePTS1 (light grey). OD_660_ values obtained after 16 h of growth of cultures containing methanol as sole carbon sources. Error bars represent the standard deviation (SD) from three independent biological replicates. **b** iEM analysis using antibodies against alcohol oxidase and catalase of cells of a WT strain producing GFP-TEV-Nsp1^1200^-ePTS1. Cells were grown on methanol for 4 h. P; peroxisome. M; mitochondrion. N; nucleus. Scale bar, 200 nm

Our data indicate that only a portion of the unfolded proteins was imported into the peroxisomal matrix. Invariably, we also detected cytosolic fluorescence when GFP-containing fusion proteins were used. Moreover, only a portion of the protein was protected against NIa in the in vivo protease protection assay. We therefore wondered whether some of the very long, unfolded fusion proteins may get stuck in the importomer and affect import of endogenous peroxisomal matrix proteins. Previous observations revealed that cytosolic mislocalization of peroxisomal matrix proteins in *H. polymorpha* results in a methanol growth defect (Gunkel et al. 2004). We therefore analyzed growth of WT cells producing GFP-TEV- Nsp1^1200^-ePTS1 or 2xHA-TEV-Nsp1^1200^-ePTS1 on medium containing methanol as sole carbon source. As shown in Fig. 5a, growth on methanol was not affected in WT cells producing any of the two fusion proteins. As expected, cells of the two control *pex5* strains producing fusion proteins were unable to grow on methanol. Normal import of endogenous proteins was confirmed by iEM analysis using antibodies raised against Aox and Cta. As shown in Fig. 5b, both proteins normally localized to peroxisomes. This shows that the unusual long, unfolded structure of the GFP-TEV-Nsp1^1200^-ePTS1 fusion protein does not affect import of endogenous peroxisomal matrix proteins.

## Discussion

Here, we show that very long, intrinsically unfolded proteins of almost 500 nm in length can entirely pass the peroxisomal importomer. Using a combination of FM, (quantitative) iEM, and an in vivo protease protection assay, we show that several fusion proteins, containing long intrinsically unfolded regions of the nuclear pore protein Nsp1, are still translocated across the peroxisomal membrane in *H. polymorpha*. The Nsp1 fusion proteins do not bind cofactors and are not capable of folding. Thus, the PTS1 at the C-terminus of the fusion protein is readily accessible from the moment it has been translated for peroxisomal matrix import.

Import of an unfolded protein into the peroxisomal matrix has not previously been reported. Peroxisomal matrix protein import receptor recycling is driven by ATP hydrolysis, which is required by the AAA-ATPase complex consisting of Pex1 and Pex6 to export Pex5 from the peroxisomal matrix to the cytosol. However, it is unlikely that this also drives the import of very long, unfolded proteins across the peroxisomal membrane into the peroxisomal matrix. The fully unfolded fusion proteins range between 408 and 480 nm in length (Bright et al. 2001; Ainavarapu et al. 2007). Conversely, the average size of a globular protein ranges between 2 and 6 nm in diameter, and a 500-kDa protein would fold into a structure of approximately 5.2 nm in diameter (Lukatsky and Shakhnovich 2008; Erickson 2009; Tiessen et al. 2012).

Current models for Pex5-cargo import propose a Pex14 grabbing mechanism, which appears to have a limited mechanistic range in terms of pulling folded proteins into the peroxisomal matrix (Skowyra et al. 2024; Gaussmann et al. 2024). This indicates that Pex13 and Pex14 must remain closely together. This limited mechanistic range works well for a folded or oligomeric protein bound in a Pex5–cargo complex. Hence, Pex5 bound to a folded protein has a much smaller physical size compared with the calculated length of our longest unfolded fusion protein (almost 500 nm). However, if the long unfolded Nsp1 is released from Pex5 too early, it would still be in the process of passing the Pex13 pore and could slip back into the cytosol. Thus, an extended pulling mechanism is required to completely import the unfolded Nsp1.

The yeast peroxisomal membrane has been determined to be more fluid and contain more disordered lipids than the plasma membrane, mitochondrial, and endoplasmic reticulum membrane (Reglinski et al. 2020; Carmichael and Schader 2022). The fluidity of the peroxisomal membrane makes it ideal for lateral movement of protein complexes by diffusion (Jacobson et al. 2019). Recent evidence suggests that Pex13 undergoes liquid–liquid phase separation in order to form a transient pore together with Pex14 for import of peroxisomal matrix proteins (Ravindran et al. 2023).

**Fig. 6:**
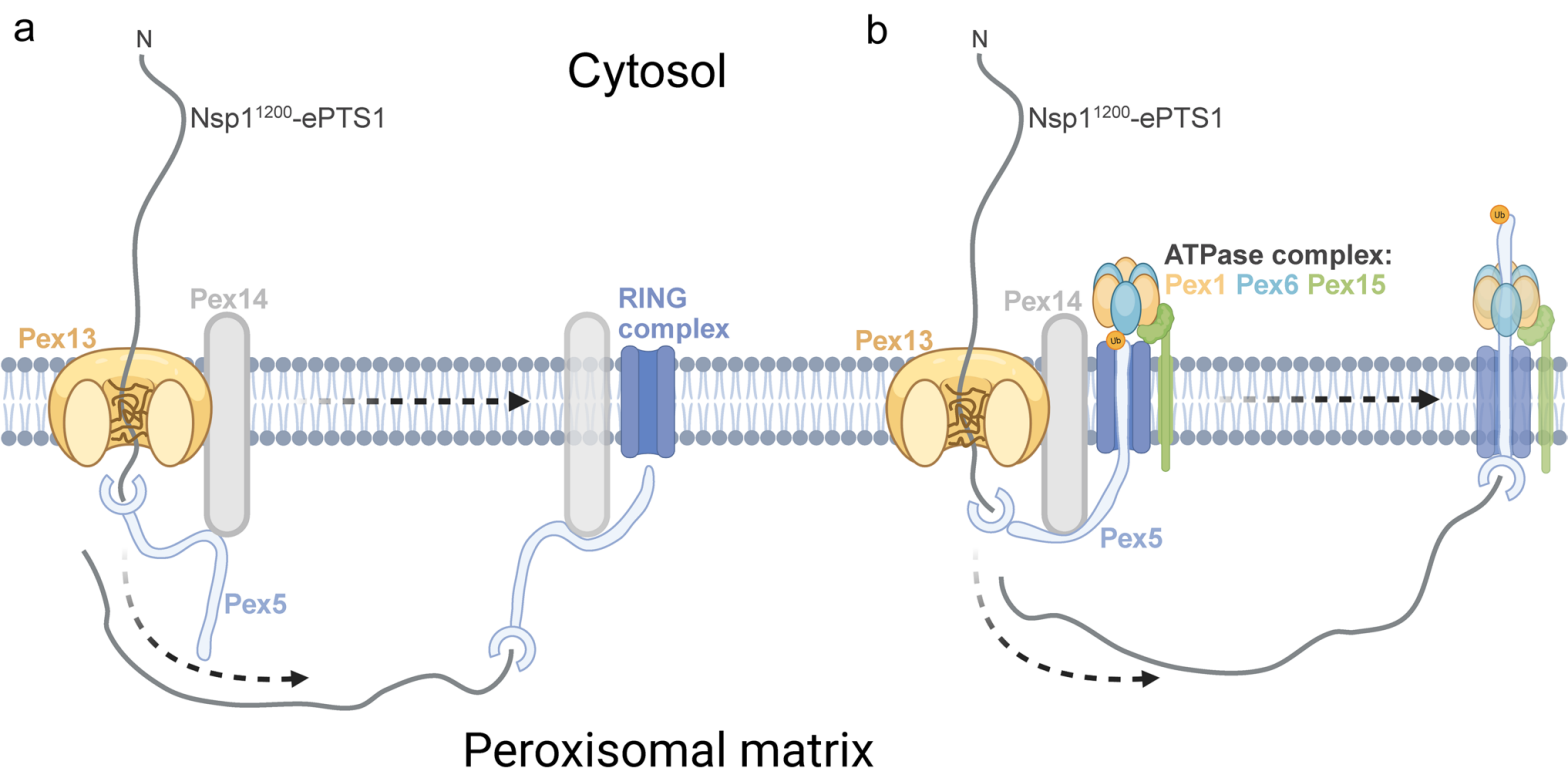
Hypothetical models of peroxisomal matrix protein import of a fully unfolded PTS1 protein. **a** Import involved dissociation of the Pex13–Pex14 complex and lateral movement of Pex14 to the ring complex. Pex5 associated to the unfolded PTS1 protein passes the Pex13 protein import pore. The FG repeats of the Nsp1 proteins may interact with the YG phase of Pex13, however the proteins ultimately pass the pore. Next Pex14 binds the Pex5–cargo complex at the peroxisomal matrix side. Upon entry of Pex5 into the peroxisomal matrix, Pex14 dissociates from Pex13 and by lateral membrane diffusion moves away from the Pex13 pore. This lateral movement causes a pull on Nsp1 which drags the long unfolded regions into the peroxisomal matrix. **b** Lateral movement of the RING complex away from the Pex13–Pex14. Pex13 and Pex14 remain bound as a complex. Upon entry of Pex5 to the peroxisomal matrix, it binds to Pex14, and the N-terminal of Pex5 binds to the RING complex and is monoubiquitinated (Ub). This binding causes Pex5-Nsp1 to be released from Pex14 and the RING complex is free to move in the membrane by lateral diffusion. Additionally, the ATPase complex consists of the membrane anchor Pex15 that tethers the two AAA-ATPases Pex1 and Pex6 to exert a pull mechanism on Ub-Pex5 for export to the cytosol. The ligase RING complex represents Pex2, Pex10, and Pex12 (blue transmembrane box). The Nsp1^1200^-ePTS1 protein is represented as an unfolded linear peptide in dark grey, and Pex5 bound at the extreme C-terminal to ePTS1. Size of proteins and membrane not to scale

One mode of import could be part of relaxation of the transient pore and reversal of the liquid–liquid phase separation to base state. In this model, upon import, Pex14 binds to Pex5(-Nsp1) on the peroxisomal matrix side (Fig. 6a). The Pex14-Pex5(-Nsp1) complex then dissociates from the Pex13 pore and laterally diffuses along the peroxisomal membrane (Gopalswamy et al. 2023; Skowyra et al. 2024) (Fig. 6a). The lateral diffusion of membrane proteins would be caused by Brownian or lipid raft movements (Carmichael and Schader 2022). Movement of Pex14 would exert a tugging mechanism on the Pex5-Nsp1 complex and pull in the remaining cytosolically located parts of Nsp1 through the Pex13 pore into the peroxisomal matrix (Fig. 6a).

However, if Pex14 remains bound to Pex13 throughout protein import, this model is not possible (Gao et al. 2022). It has previously been proposed that the peroxisomal matrix protein import complex consisting of Pex13, Pex14, and the RING complex consisting of Pex2, Pex10, and Pex12 could form a supercomplex in the membrane. However, recent evidence suggests that the Pex13–Pex14 import complex and the RING complex are two separate structures (Skowyra et al. 2022, Feng et al. 2022a, b; Gao et al. 2022; Ravindran et al. 2023).

In the model in Fig. 6b, the AAA-ATPase complex consisting of Pex1/Pex6 (which exports Pex5) exerts a pull on Pex5-Nsp1 for import. The export of Pex5 through Pex1/Pex6 would further drag the unfolded Nsp1 protein into the peroxisomal matrix (Fig. 6b). Additionally, the RING complex could also contribute to the pulling mechanism for Pex5-Nsp1, but given the size of our unfolded proteins, other as yet unknown mechanisms are likely to be involved as well (Fig. 6b).

The *H. polymorpha* Pex5 is 569 aa long, wherein residues 272aa–517aa form the tetratricopeptide repeat (TPR) structure responsible for binding to PTS1 (*S. cerevisiae* Pex5 612aa) (van der Leij et al. 1993; van der Klei et al. 1995; Skowyra et al. 2022). This means that a Pex1/6 pull mechanism can only displace 272aa of our unfolded protein (1247aa) into the peroxisomal matrix before the TPR domains of Pex5 reach the rigid export pore in the RING complex. At the RING complex, the Pex5 TPR would unfold, releasing the unfolded protein in the peroxisomal matrix prior to full import (Feng et al. 2022a, b). This only leaves 22% of our unfolded protein through the Pex13 pore but 975aa remaining on the cytosolic side of the peroxisomal membrane. Thus, the model represented in Fig. 6 would still require rapid lateral diffusion of the RING/ATPase complex while bound to Pex5-Nsp1 away from the Pex13 pore, otherwise Pex5 would be exported and released prior to full import of our unfolded protein.

## Conclusions

This study investigated whether a relatively long, unfolded protein could be imported to peroxisomes in the yeast *H. polymorpha*. Through FM, (quantitative) iEM, and an in vivo protease protection assay, we found that a completely unfolded protein consisting of 1247 residues can be fully imported into the peroxisomal matrix. Current models for peroxisomal matrix protein import only account for folded and oligomeric proteins. Here, we propose two possible lateral pull mechanisms based on membrane protein complex dissociations to drag a long unfolded polypeptide into the peroxisomal matrix. The first model proposes the dissociation between the import pore Pex13 and the membrane protein Pex14 to drag in unfolded protein. Here, Pex14 is bound to Pex5-Nsp1. In the second model, the membrane RING/ATPase complex dissociates from Pex13–Pex14, which drags the unfolded protein into the peroxisomal matrix. Here, Pex5–Nsp1 is bound to the RING/ATPase complex. Although the Pex1–Pex6 ATPase complex exerts an additional pull mechanism on Pex5-Nsp1 owing to its recycling function of the PTS1 receptor, an additional as yet unknown mechanisms for complete import most likely exist.

## Supplementary Information

Below is the link to the electronic supplementary material.

Supplementary file1 (XLSX 16 KB)

## Data Availability

No datasets were generated or analyzed during the current study.
